# Paradoxical supine overdrainage with ventriculoatrial shunt

**DOI:** 10.1186/2045-8118-12-S1-P50

**Published:** 2015-09-18

**Authors:** David Solomon, Abhay Moghekar, Ari Blitz

**Affiliations:** 1 Johns Hopkins University, USA

## Introduction

Antisiphoning devices and gravitational valves (ShuntAssistant) have been used successfully to decrease overdrainage of CSF due to postural change, by limiting the amount of CSF outflow in the upright position. The possible advantage of a VA configuration conferred by a smaller hydrostatic column is lessened by the lack of a longer, smaller diameter distal catheter. We observed subatmospheric intracranial pressure in supine patients with ventriculoatrial shunt, associated with headaches that were worse lying down and upon awakening in the morning.

## Methods

Patients in this case series had ventriculoatrial shunting for pseudotumor cerebri, with a ShuntAssistant in series with a programmable differential valve. Intracranial pressure was determined by measuring CSF pressure through a lumbar spinal catheter with the fluid-coupled transducer at the level of the external auditory meatus, after zeroing to atmospheric pressure. Respiration was measured with impedance plethysmography. Data were continuously sampled at 100 Hz and stored for offline analysis.

## Results

CSF pressure gradually decreased to below zero when patients' position changed from upright to lying down. Supine pressures were often lower than when patients were sitting upright, opposite to the usual situation in both shunted and physiologic conditions. Mean CSF pressure could be between -5 and -10 mm Hg for hours, with the amplitude of respiratory modulation of the ICP waveform as great as 15 mm Hg.

Contrast-enhanced brain MRI did not show pachymeningeal enhancement or dural thickening, but often showed asymmetric ventricles consistent with overdrainage.

## Conclusions

The usual mechanism for ventricular shunt overdrainage – hydrostatic pressure due to fluid in the distal catheter- cannot explain these observations. Negative inspiratory intrathoracic pressure associated with obstructed breathing is a likely cause, given the large respiratory modulation of the pressure waveform. Aspiration due to blood flow around the distal catheter tip is an additional possible mechanism, especially since there is increased venous return to the heart during inspiration. We suggest that negative ICP in the supine patient is due to a respiration-generated pressure gradient favoring venous outflow through the cerebral sinuses, and CSF outflow through the differential valve, with no added resistance from the additional gravitational valve.

Headaches worse when lying and present upon awakening in the morning may be mistakenly attributed to elevated ICP, prompting adjustment to a lower valve opening pressure. When patients with VA shunt have labored breathing and some degree of ventricular collapse, abnormally low ICP due to paradoxical supine overdrainage should be considered.

## References

[B1] Radvany MG Solomon D Nijjar S Subramanian PS Miller NR Rigamonti D Blitz A Gailloud P Moghekar A Visual and neurological outcomes following endovascular stenting for pseudotumor cerebri associated with transverse sinus stenosis J Neuroophthalmol 2013 33 2 117 22 10.1097/WNO.0b013e31827f18eb 23502837

[B2] Xie YJ Shargorodsky J Lane AP Ishii M Solomon D Moghekar AM Gallia GL Douglas D Reh DD Perioperative Continuous Cerebrospinal Fluid Pressure Monitoring in Patients with Spontaneous Cerebrospinal Fluid Leaks Int Forum Allergy Rhinol 2015 5 1 71 7 doi: 10.1002/alr.21424. Epub 2014 Oct 2 10.1002/alr.21424 25278379

